# Supplementary material for: Complex marine ecological response during the Eocene-Oligocene revealed by global foraminiferal record
Source: Nat Commun. 2026 Mar 14;17:3954. doi: 10.1038/s41467-026-70541-w (PMC13133226; doi:10.1038/s41467-026-70541-w)
Supplement: Supplementary file 1 — Supplementary Information [file 41467_2026_70541_MOESM1_ESM.pdf]

Supplementary Information for
Complex marine ecological response during the Eocene-Oligocene revealed by global foraminiferal record

Zhengbo Lu (鲁铮博)[1,2†], Ke Xue (薛轲)[3,4†], Yiying Deng (邓怡颖)[5†], Junxuan Fan (樊隽轩)[1,2✉], Peiyue Fang (方培岳)[6], Bridget S. Wade[7], Laia Alegret[8], Michael J. Benton[9], Yuchang Wu (吴宇畅)[3,4], Chao Qian (钱超)[3,4✉], Xudong Hou (侯旭东)[1,2], Yukun Shi (史宇坤)[1,2✉], Peter M. Sadler[10], Huiqing Xu (徐卉清)[1,2], Zhi-Hua Zhou (周志华)[3,4], Shuzhong Shen (沈树忠)[1,2]

[1]State Key Laboratory of Critical Earth Material Cycling and Mineral Deposits, School of Earth Sciences and Engineering, Nanjing University, Nanjing, 210023, China.
[2]Frontiers Science Center for Critical Earth Material Cycling, Nanjing University, Nanjing, 210023, China.
[3]National Key Laboratory for Novel Software Technology, Nanjing University, Nanjing, 210023, China.
[4]School of Artificial Intelligence, Nanjing University, Nanjing, 210023, China.
[5]School of Resources and Environmental Engineering, Hefei University of Technology, Hefei, 230009, China.
[6]School of Earth and Planetary Sciences, East China University of Technology, Nanchang 330013, China
[7]Department of Earth Sciences, University College London, Gower Street, London, WC1E 6BT, UK.
[8]Department of Earth Sciences, University of Zaragoza, Zaragoza, 50009, Spain.
[9]School of Earth Sciences, Wills Memorial Building, University of Bristol, Bristol, BS8 1RJ, UK.
[10]Department of Earth Sciences, University of California, Riverside, CA 92521, USA.

[†]These authors contributed equally: Zhengbo Lu, Ke Xue, Yiying Deng.

✉e-mail: jxfan@nju.edu.cn; qianc@nju.edu.cn; ykshi@nju.edu.cn

This PDF file includes:

Supplementary Notes
Figures S1 to S9
Tables S1 to S4
SI References

Supplementary Notes

Supplementary Note 1: Basic introduction to foraminifera and their ecology

Foraminifera are broadly classified into three groups: planktonic foraminifera (PF), larger benthic foraminifera (LBF), and small benthic foraminifera (SBF). Each group exhibits unique responses to various environmental drivers[1,2].

PF are primarily found in open ocean waters. They are significantly influenced by sea surface temperatures, as temperature plays a crucial role in their survival and geographical distribution[2,3]. Moreover, PF are indicators of past sea surface temperatures, with their taxonomic and isotopic compositions providing valuable data for climate reconstructions[4,5]. Nutrient availability and light intensity also play significant roles, particularly in shaping their vertical distribution in the water column and impacting their symbiotic relationships with photosynthetic organisms[6]. Additionally, oxygen levels and salinity are important for PF[7,8], with variations in these factors influencing their growth and survival.

LBF predominantly inhabit tropical and subtropical shallow marine environments. Their distribution is more geographically and ecologically restricted compared to SBF, as they prefer to live in warm conditions[9,10]. Light intensity and nutrient availability are key factors affecting LBF, particularly due to their phototrophic symbiotic relationships[11,12]. LBF are also sensitive to changes in salinity[13,14], which can affect their growth and survival.

SBF are versatile, inhabiting a range of depths from shallow to deep marine environments. They are less restricted in distribution than LBF. Factors such as dissolved oxygen levels and food supply play an important role in their survival[10], with fluctuations in oxygen levels and the interaction between oxygen-food supply linked to many historical extinction events of foraminifera.

Supplementary Note 2: Dataset overview and standardisation of the stratigraphic and paleontological data

All the data were manually collected from published literature by the OneStratigraphy team and stored in the OneStratigraphy Database (http://onestratigraphy.ddeworld.org/). To obtain a complete view of the foraminiferal records in OneStratigraphy, users are recommended to follow these steps: Navigate to Stratigraphic Data → Advanced Search → Occurrences and set the fossil group to

Foraminifera. As of 12 June 2025, the OneStratigraphy Database contained 826,688 foraminiferal occurrences.

Each stratigraphic section is assigned a unique Section ID in OneStratigraphy (Supplementary Data 2), allowing direct traceability of data entry history, contributor identity, and original literature sources. For example, the first and last entered entries in Supplementary Data 2 correspond to the sections Zongpubei (Section ID: SE202005060304540) and Alano (Section ID: SE202110280146975), respectively. Querying these Section IDs in OneStratigraphy (Stratigraphic Data → Advanced Search → Section → Section no.) retrieves the full section records. The Zongpubei section was digitised and entered into OneStratigraphy by Qin Chen on 6 May 2020, while the Alano section was digitised and entered by Xiaohong Zhou on 28 Oct. 2021. In both cases, the original literature sources from which the stratigraphic information was digitised are documented in the View reference tab associated with each section record. The related information of bibliography, data enterer and date of entry can be found in Supplementary Data 2. This structure ensures transparent and clear linkage of all input sections to their original published sources within the OneStratigraphy Database. Additionally, any errors found in the process were corrected (e.g., spelling mistakes in species names) and missing information was filled in (e.g., latitude and longitude data).

We selected sections/sites containing foraminifera occurrences from the Eocene to the Oligocene. The raw dataset contained 13,138 local bioevents records (i.e., first and last appearance records) and ~60,000 occurrences of 2,988 taxonomic units from 163 published stratigraphic sections, encompassing both calcareous and agglutinated foraminifera. These sections, including drill cores and outcrops, are widely distributed across modern oceans and continents such as Europe, Africa and Asia (Fig. S4).

The dataset was first cleaned by excluding open nomenclature, such as sp./spp. (622), aff. (63), question marks for species names (6). Nevertheless, the conferring species (cf.; 175) and the group species (ex gr.; 25) were preserved and assigned to the referenced species. Taxonomic assignments below the species level (i.e., subspecies and variety) were mostly integrated to species level. All non-foraminifera fossils were removed. The dataset after cleaning was thoroughly examined and verified against other independent data sources, including taxonomic atlases[15,16], foraminiferal databases (Mikrotax and WoRMS), and related taxonomic references, and further verified and resolved by a group of foraminiferal taxonomic experts for correctness and consistency: Bridget Wade (PF), Laia Alegret (SBF), Qinghai Zhang (LBF and SBF), and Peiyue Fang (PF, LBF and SBF).

In the present dataset, the drill core Hole 647A has been studied repeatedly within a comparable stratigraphic framework, focusing on both SBF and PF for variable purposes, such as testing biotic response to EOT, studying high-latitude deep-water sedimentary sequence, and stratigraphic correlation[17-19]. The three reports[17-19] were integrated into one section by depth.

The final dataset after data cleaning and verification included ~40,000 fossil occurrences and 9,032 local first and last occurrence records of 1,269 species in 161 published stratigraphic records (drill cores and outcrops).

All original data are available as section records on the online platform of the OneStratigraphy Database. The final dataset (as Excel file and CONOP data files) after data cleaning and verification, as well as the CONOP program package, are also available as a zip file for free download from the OneStratigraphy (https://onestratigraphy.ddeworld.org/download/0f53e05eed3243f1898dc5afd2c54134) or Dryad (https://doi.org/10.5061/dryad.jh9w0vtk5) repositories.

Supplementary Note 3: Network analysis in stratigraphic correlation among sections

Network analysis was applied to the present dataset by using Gephi software. The result demonstrates the section relations of the study dataset (Fig. S5). Nodes and edges in the non-directed network represent the sections and shared taxa among sections, respectively. The weights of edges are twice the number of shared taxa divided by the sum of taxa in linked sections, which represents the similarity of fossil composition among sections. The network analysis result shows that all the sections in the study dataset are tightly linked through shared species (Fig. S5; The layout of the network is based on the Fruchterman Reingold algorithm[20]). Node centrality, shown by node size, is estimated by node degrees, i.e., the sum of edges of the node combined with the edges' weights, indicating the importance of sections in stratigraphic correlation. The strong connectivity across the dataset supports its suitability for subsequent CONOP-based stratigraphic correlation, as all sections/sites are biostratigraphically linked. Furthermore, the nodes (sections) in the network were grouped into five communities (Modularity = 0.419) by using the fast unfolding of communities method[21] and shown in different colours in Fig. S5. These communities can be interpreted as approximations of biogeographic provinces[22]. Nodes within each community share similar fossil assemblages, reflecting comparable palaeogeographic and stratigraphic relationships. While this observation opens opportunities for future research into biogeographic

connectivity using our high-resolution dataset, such analyses are beyond the scope of the present study.

Supplementary Note 4: Calculation and use of virtual sequence

When the size of the study dataset becomes larger, i.e., the numbers of sections or taxa increase, the requirement for computing power will increase as well. One of the possible reasons is the demand for huge trials in the face of exponentially increasing solution space (i.e., possible sequences) depending on the complexity of the dataset[23]. The cost on the supercomputer is about 1–2 cents / (core × hour), so that the cost for only one calculation on the dataset in Fan et al.[24] could be over $10,000. Therefore, a virtual section technique was adopted to speed up the calculation, which helps the program reach the proper solution space quickly and greatly saves the calculating resource.

The virtual section is composed of planktonic foraminiferal index fossils and magnetochrons. The order of their first and last appearance levels, which we refer to as the virtual sequence, comes from widely accepted stratigraphic standards such as GTS 2020[25,26] and Wade et al.[27]. The order of these fossils was further evaluated by running CONOP on a small subset of the study dataset which only contains sections with these index fossils. Magnetochrons are tested in five GSSP and GSSP candidate sections containing them for the consistency with index fossils. If there are any inconsistencies between the CONOP results/real sections and the virtual sequence, we should refer back to the original stratigraphic report to determine the source of the problem. In our experience, inconsistencies mainly arise from incompleteness of fossil ranges in sections (even in GSSP sections). For example, the last appearance of *Catapsydrax dissimilis* in Hole 1130A&C occurs at a lower level than that recognised in the planktonic foraminifera zonation[27]. Checking the original publication reveals that this site has a low recovery, with the species *C. dissimilis* only found in one sample and being rare. Therefore, the virtual sequence after verification is robust not only in previous studies but also in the present dataset (Table S2).

Further analysis without any weighted virtual sections was conducted to test the validity of the virtual sequence and estimate the cost for acceptable optimised results. A subset of the study dataset was explored following the criteria: (1) the sections were densely collected and cover a considerably longer stratigraphic interval; (2) the sections contain diverse foraminifera, especially those index fossils; (3) the subset is big enough, e.g., containing about 1/3 to 1/4 species of the whole dataset, so that a relatively convincing analysis can be done at a reasonable cost. The generated subset, which

contains 308 species from 12 sections, was subsequently calculated by varying trials but constant start temperature (500) and steps (700). It should be noted that none of the sections or taxa is weighted. The subset was calculated ten times for each set of parameters, and the consistency index was calculated to depict the consistency of the virtual sequence and the order of those first or last appearance levels in each result. It shows that consistency increases with exponentially increasing trials (r^2 = 0.98; Fig. S6), indicating the validity of the virtual sequence and that the reliability of the result sequence depends on the number of trials at exploring the solution space. However, the original dataset contains about four times more species and nearly 15 times more sections than the experimental subset, thus it will require an inestimable number of trials to obtain an optimised sequence for the original dataset, which is undoubtedly a huge cost.

The verified sequence of index fossils (Table S2) and magnetochrons (tested in five GSSP and GSSP candidate sections containing them) was used to construct a virtual section, which was included in the study dataset and highly weighted in the subsequent CONOP computations to speed up the calculations on the supercomputer. CONOP.SAGA was first run on the Tianhe II supercomputer ten times with the following parameters: starting temperature = 500, steps = 700, trials = 2,400,000 or 4,800,000. The Tianhe II supercomputer comprises 16,000 compute nodes, each equipped with two Intel Xeon E5-2692 v2 12-core CPUs operating at a base clock of 2.2 GHz (turbo up to 3.0 GHz) and three Intel Xeon Phi 31S1P co-processors—yielding a total of 3,120,000 CPU cores and 48,000 coprocessor cards—backed by 1,375 TiB of DDR3 system memory and a 12.4 PB global storage system. Our project, requesting 4 nodes (256 CPU cores), was provisioned with 1 TB of user file-system space. Ten results were collected and loaded in CONOP.EA. The program used them as parent sequences to generate 10 offspring sequences for an initiating population, which then evolved one generation to another. The CONOP.EA program collected all the optimised sequences and saved them in a library of optimised sequences, most of which were better than their parent sequences generated by CONOP.SAGA. The most optimal sequence was chosen for subsequent richness analyses (Fig. S7).

Supplementary Note 5: Calibration of the composite sequence to the geologic time scale

Due to the lack of sufficient high-precision isotope dating for the study interval, the composite sequence acquired from CONOP.EA was ultimately calibrated to GTS

2020[25,26] by using geological ages corresponding to the boundaries of seven planktonic foraminifera zones near the stage boundaries and seven well-identified and robust magnetochrons (Fig. S8 and Table S4).

In this study, the age model was constructed by fitting a cross-validated smoothing spline to the tie points (biozones and magnetochrons), selecting its smoothing parameter by leaving-one-out cross-validation to achieve an optimal trade-off between fidelity to the data and overall smoothness[28]. The 95% confidence interval was quantified by 1000 residual bootstrap iterations. Smoothing splines do not enforce exact interpolation at every tie-point (assumption of isochrony) but instead balance goodness-of-fit against overall smoothness, so the resulting curve may deviate slightly from individual tie-points to achieve a globally optimal solution. For instance, the tie-point for the last common occurrence (LCO) of *Chiloguembelina cubensis* is allowed to shift modestly, since its empirical common occurrences lie a little below the highest last occurrence recorded in the CONOP-derived composite sequence.

Supplementary Note 6: Construction of unbinned richness curve and its standardisation

After acquiring a calibrated composite sequence with 962 discrete temporal levels, an unbinned method was used to generate a species-level richness curve (Fig. 2a). Richness was initially set at 0, and if there was a first appearance in the next temporal level, the richness value was increased by one. If there was a last appearance, the richness value was decreased by one. This unbinned method can preserve the original high temporal resolution and avoid bias due to uneven subdivision of time bins. The same method was also applied to generate the genus-level richness curve by tracking generic changes through the composite sequence (Fig. 2a). The species/genus ratio was easily calculated for each temporal level by dividing the species richness by the genus richness (Fig. 2a). The constructed species richness curves were comparable to those of previous studies (Fig. S2).

Sampling effect is inevitable when generating richness curves. Many researchers had put forward concerns that strong sampling biases may influence the accuracy of the curve[29-33]. In addition to the common factors that may lead to these biases, such as sampling intensity and fossil preservation, taxonomic identification plays a vital role in species-level richness studies. These studies require taxonomic data at the species level. Consequently, fossils classified at a higher taxonomic level (e.g., genus) are excluded from the dataset. This exclusion is significant for SBF, a highly diverse and disparate

group. Many SBF studies do not identify rare morphological species to the species level or include informal groupings of rare taxa. These factors collectively complicate species-level richness estimates, leading to uneven sampling sizes throughout the research interval. As a result, the absolute richness values represent an estimate of the compiled dataset, while the trend of richness changes matters if not strongly influenced by sampling biases.

Here, rarefaction was applied to test the sampling effect of our dataset by making a standardised comparison of the unevenness of the species record[34,35] in the full foraminiferal curve (Fig. 2c) and curves of three foraminiferal groups (Fig. S9), respectively. Sample size was generally uneven for different temporal levels, which might strongly influence the estimation of species richness. Taxonomic rarefaction was used to standardise the total number of local taxonomic ranges through each temporal level in the composite sequence. Take the full foraminifera curve as an example. Firstly, the sample size should be set for rarefaction based on sample size distribution throughout the study period (from 48 Ma to 20 Ma). The smallest sample size of the study period is 626, so the sample size of each temporal level can be standardised to 300 and 500, respectively. Next, a simulated sample is set in each level based on the taxa ranges and their section distribution, then the species at each level are resampled without replacement, and then the rarefied species richness is calculated (Fig. 2c). Although the changes of the rarefied curves were less remarkable than the unrarefied curve, the major changes were still identifiable, confirming that sample size did not significantly influence the estimation of the main richness trajectories in the present dataset. The same method was applied to the three groups of foraminifera (Fig. S9). Sample size at each time level was standardised to 50, ensuring that richness estimates were fair and comparable among intervals with different degrees of sampling bias. This process was iterated 1000 times to generate a robust estimate of rarefied richness. The results indicated that richness trends show no remarkable changes, though the amplitude of some events (e.g., SBF in RRD) was reduced. Therefore, the richness trend estimate is robust, indicating reliable richness changes during the EOT.

Supplementary Note 7: Species richness change rate

A binned method was used to calculate the richness change rates including proportional origination, extinction, turnover and diversification rates[36,37] over a uniform time bin of 0.2 Myr. The 0.2 Myr bin size was chosen as the bin duration because the temporal resolution is uneven through the study interval, e.g., the imputed

temporal resolution of the Priabonian is ~12.7 Kyr and that of the Chattian is ~52.6 Kyr. Significant changes in these curves (Fig. 2d–g, Fig. S3d–f), together with those of the richness curves (Fig. 2a–c, Fig. S3a–c), help us identify major bioevents (e.g., extinction) through the study period. When calculating proportional origination rates, the number of first appearances of species was counted in each bin, and then divided by the duration of the time bin (i.e., 0.2 Myr) and total species richness of the bin for standardisation (Fig. 2d). The extinction rate was generated through a similar procedure (Fig. 2e). The proportional turnover and diversification rates were calculated by adding proportional origination and extinction rates, and detracting proportional origination rate by proportional extinction rate, respectively (Fig. 2f, g).

Supplementary Note 8: Potential CCD influence on SBF richness estimation

The present dataset encompasses both calcareous and agglutinated foraminifera. Therefore, fluctuations in the Calcite Compensation Depth (CCD) are likely to affect the preservation of calcareous foraminiferal fossils, thereby influencing the recorded richness of small benthic foraminifera. However, our richness curve for SBF (Fig. 3a) shows no association with documented CCD shoaling and/or deepening events (e.g., CAE 2-7) during the middle to late Eocene, as reported by Palike et al.[38]. Intriguingly, SBF richness declined during the EOT, a period characterised by a reverse deepening of the CCD. Therefore, changes in the CCD are inferred to have little influence on our results.

Supplementary Note 9: Correlation analysis of species richness and environmental proxies

The Cenozoic is currently the best time for precise correlation analysis between biodiversity history and environmental changes because many high-resolution environmental proxies have been compiled during the past decade, such as Cenozoic carbon and oxygen isotopes[39], sea level[40], sea-surface temperature[41] and deep-sea temperature[42]. However, the $\delta^{18}O_{benthic}$ is not used for correlation, as its variance inflation factor (VIF) is larger than 10, indicating potential multicollinearity with other proxies, such as sea level. Their correlations with species richness were analysed by Spearman's rank correlation. Nevertheless, some of those proxy data were published at different times and thus were calibrated to different time scales, such as GTS 2004 and 2012. Before the correlation analysis, they were calibrated to GTS 2020, the same time

scale as the present richness data. Considering the temporal resolutions of all these time series, a 0.2 Myr time bin was used to standardise both biotic and abiotic data.

The sea-level, sea-surface temperature, and deep-sea temperature time series were originally reported on earlier Geologic Time Scale versions (GTS2004 and GTS2012) and were harmonised to GTS2020 by linear interpolation between published magnetochron and/or stage tie points. The carbon and oxygen isotopic data[39], calibrated to La2010b (an orbital solution referenced in GTS 2020), required no further adjustment to align with the biotic data.

Spearman's rank correlation analysis was applied to the time series of the same time scale and also the detrended time series (Table 1), as the former shows the long-term trend and the latter focuses the interval-to-interval changes[43]. To quantify uncertainty, we estimated 95% confidence intervals for Spearman's ρ using bootstrap resampling (10,000 iterations), recalculating ρ for each resample and taking the 2.5th and 97.5th percentiles of the bootstrap distribution. There are two major methods of detrending: removing the best linear fitting line of the time series or to first-difference neighbouring values throughout the time series. Actually, detrending aims to remove the main (long-term) component of the signal and compare residual variability; however, this can inadvertently amplify high-frequency noise when individual observations carry non-negligible uncertainty, which are probably difficult in palaeo-time series, such as diversity[44] and the results of CONOP, because of the uncertainty of the heuristic algorithm (Simulated annealing). Therefore, a less severe detrending by simple linear regression is used to reduce the influence of the primary trend without over-emphasising high-frequency noise.

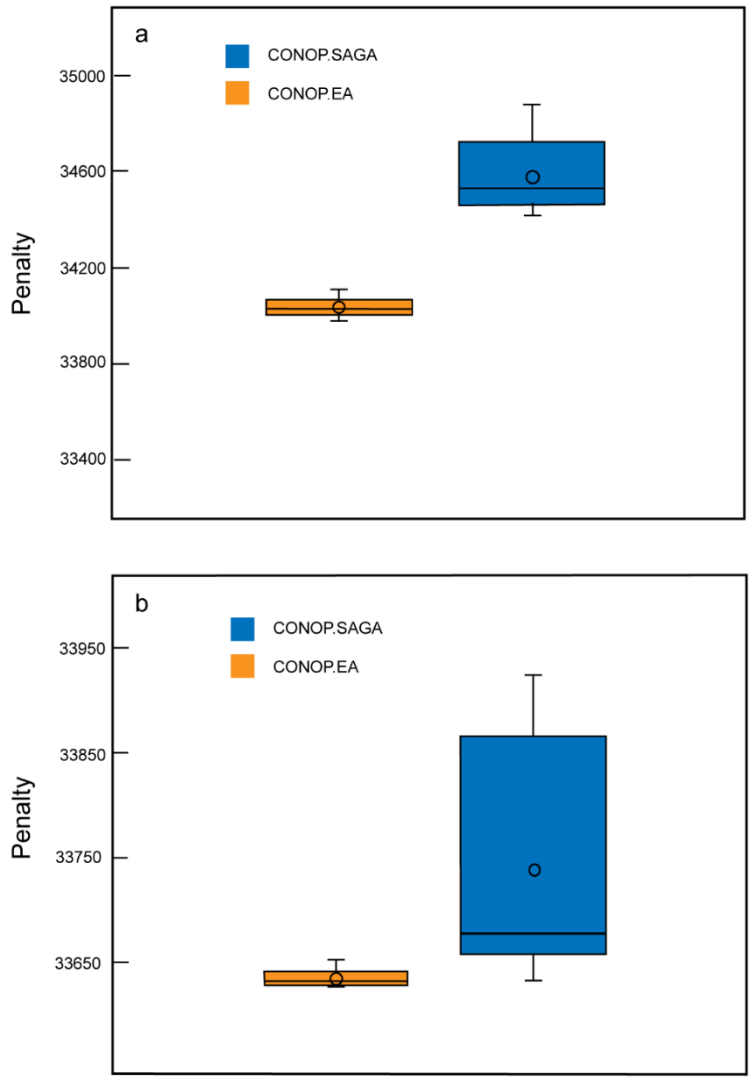

Fig. S1 | Comparison of penalties of results constructed by CONOP.SAGA and CONOP.EA, respectively. a, The two programs were applied to the present dataset with the same running parameters: starting temperature = 500, steps = 700, trials = 40,000 (n = 11 independent runs per program). b, The two programs were run in different ways. Parameters of CONOP.SAGA: starting temperature = 500, steps = 700, trials = 2,400,000 or 4,800,000 (n = 10 runs). The CONOP.EA calculation was based on ten results generated by CONOP.SAGA, and the parameters were set as: starting temperature = 0, steps = 500-700, trials = 240,000 (n = 10 runs). Circles and solid lines represent means and medians, respectively. Box plots show the median (centre line) and interquartile range (IQR; box, 25th–75th percentiles); whiskers extend to 1.5×IQR. Open circles indicate the mean.

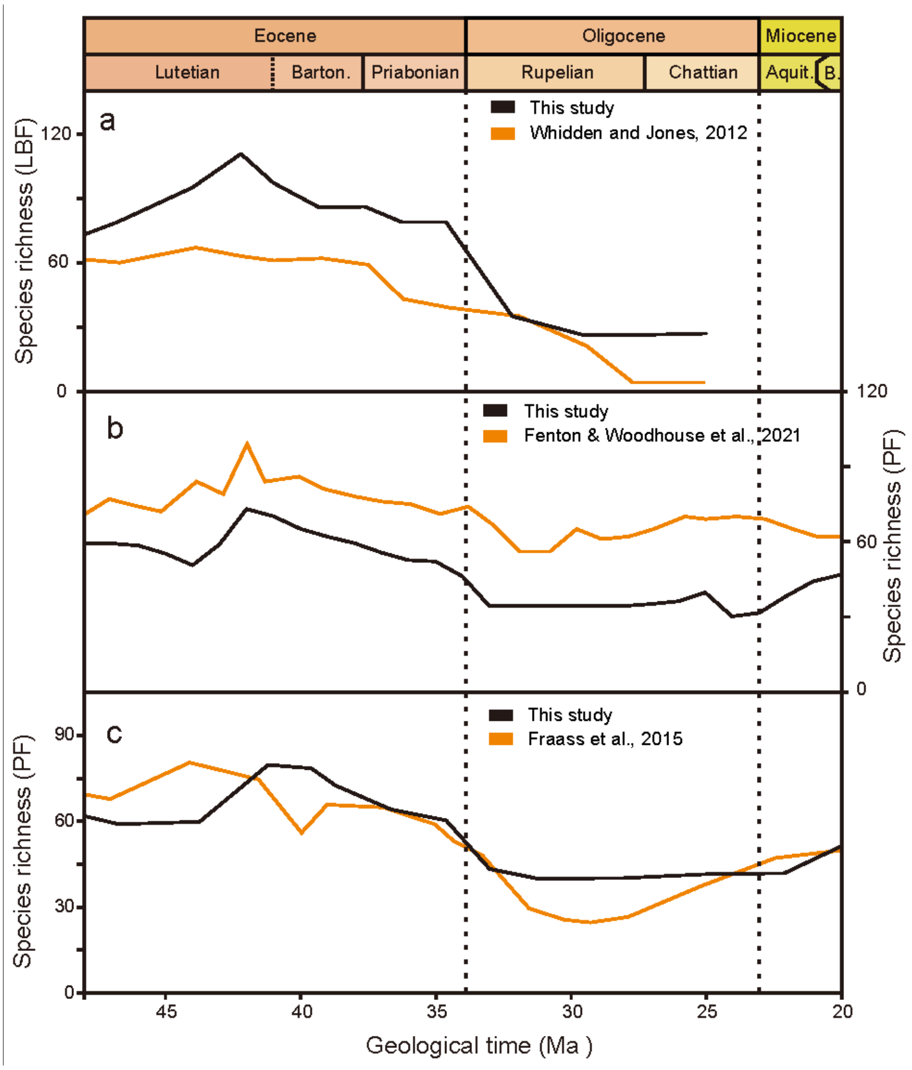

Fig. S2 | Species richness of larger benthic (a) and planktonic (b-c) foraminifera compared with those of previous studies. The curves of the present study were calibrated to the same resolution and time scale as the previous studies.

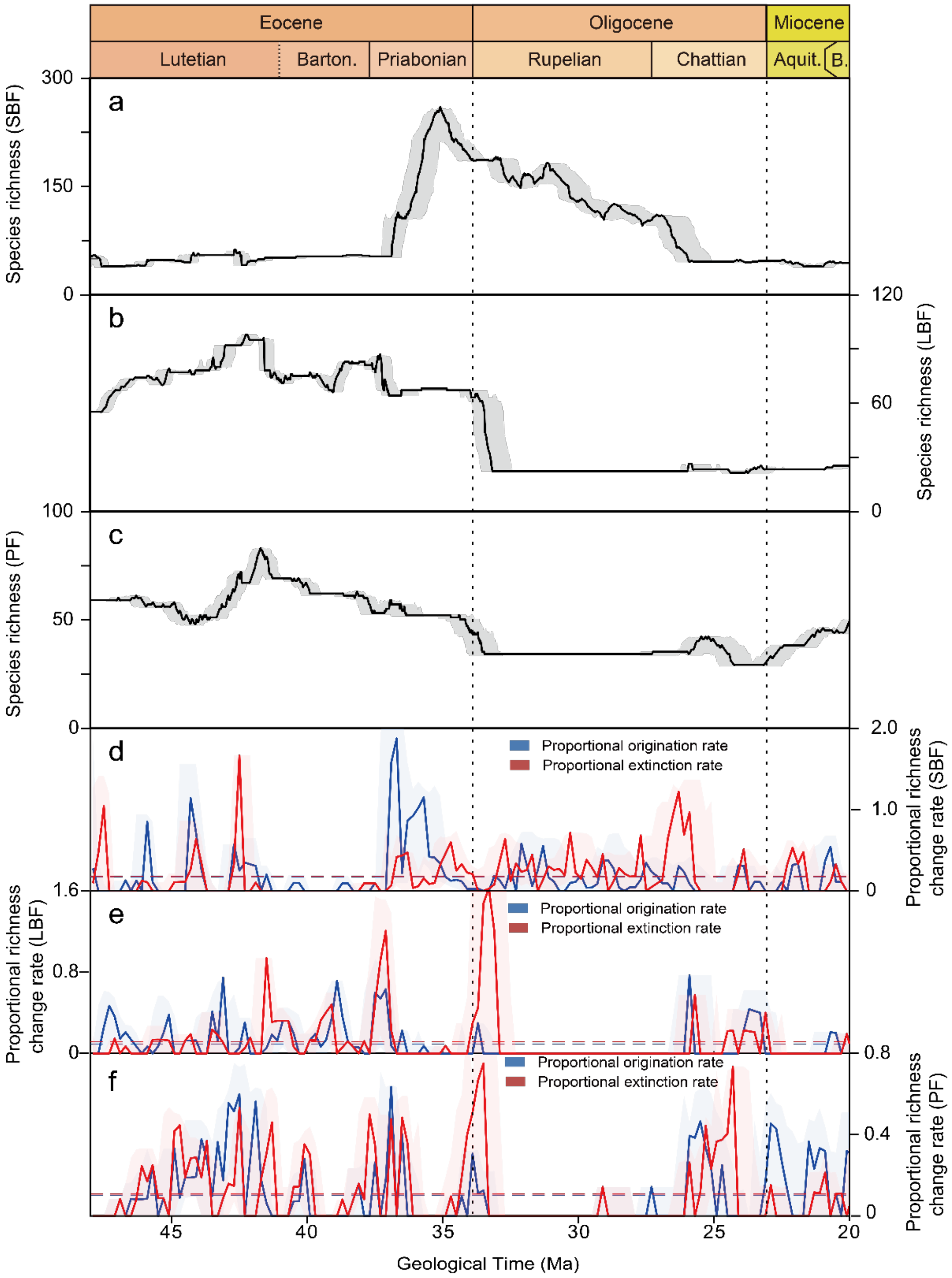

Fig. S3 | Species richness and richness change rates of foraminifera. a, Species richness of small benthic foraminifera. b, Species richness of larger benthic foraminifera. c, Species richness of planktonic foraminifera. d, Proportional richness change rates of small benthic foraminifera. e, Proportional richness change rates of larger benthic foraminifera. f, Proportional richness change rates of planktonic foraminifera. Dotted lines in d–f represent the averages. Solid lines represent point

estimates from the original data. The shadings represent the 95% confidence interval from 1,000 bootstrap iterations.

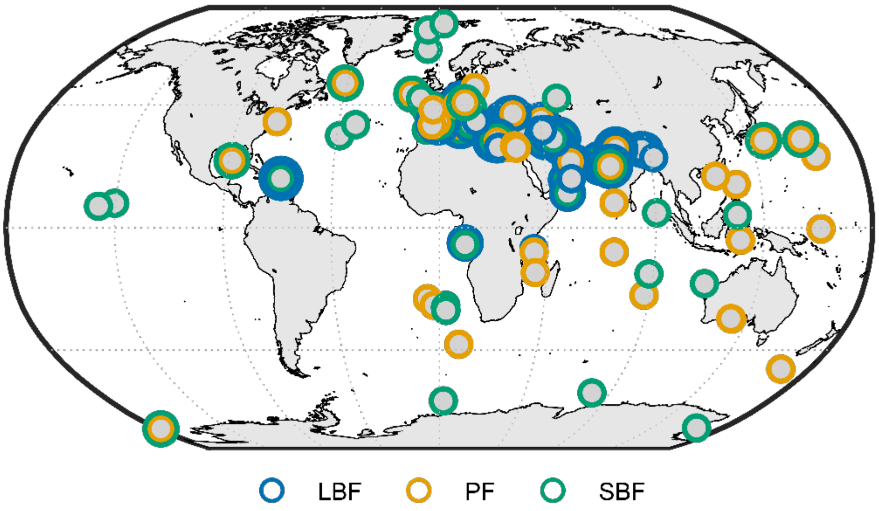

Fig. S4 | Distribution of study sections on the modern map. Blue circle represents the site/section contains occurrences of LBF. Yellow circle represents site/section contains occurrences of PF. Green circle represents site/section contains occurrences of SBF. Base map boundaries/coastlines were generated in MATLAB using the Mapping Toolbox dataset landareas.shp.

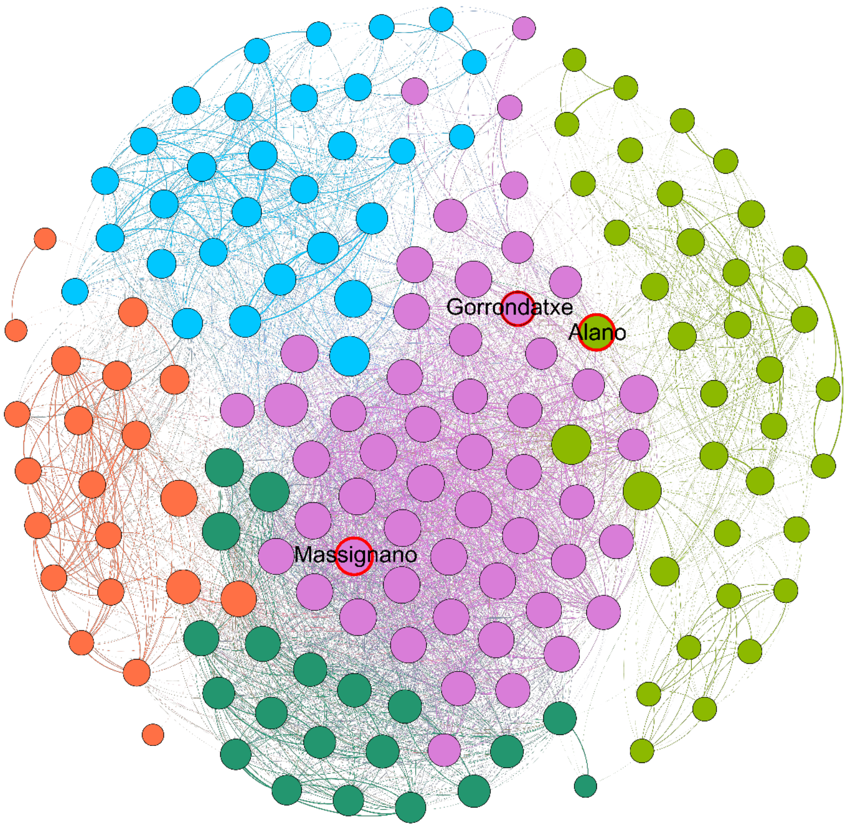

Fig. S5 | Network analysis on the study dataset, where nodes are sections and edges are the shared taxa among sections. Node sizes are their centrality of Degree. Nodes in the same colour belong to the same community identified[21]. These communities, displayed in different colours in the figure, can be interpreted as approximations of biogeographic provinces[22]. Nodes within each community share similar fossil assemblages, reflecting comparable paleogeographic and stratigraphic relationships. The layout of the network is based on the Fruchterman Reingold algorithm[20]. The names labelled in the network refer to the three GSSPs included in the study dataset.

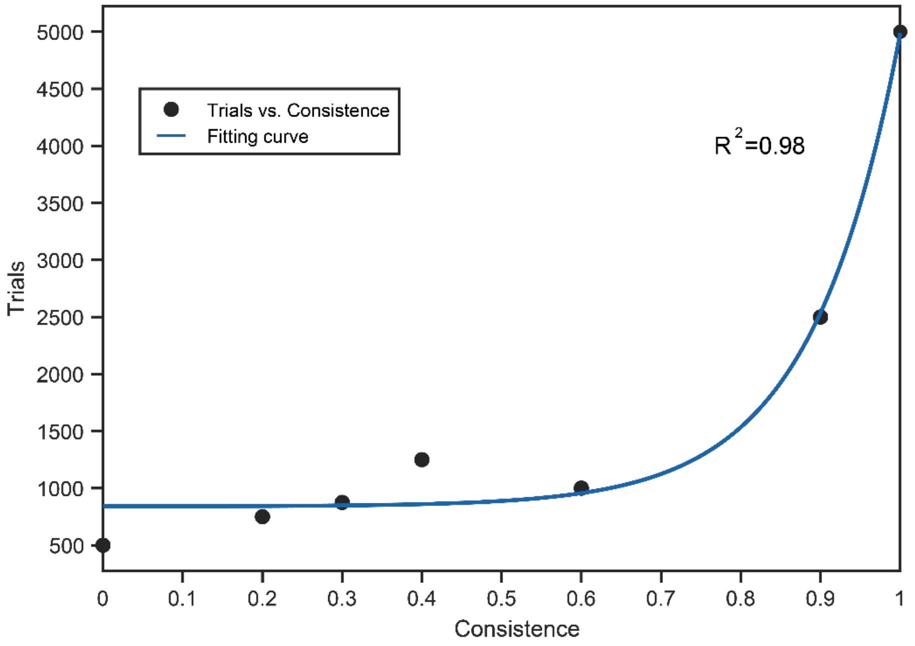

Fig. S6 | Correlation showing the relationship between the calculation cost and consistency of the result sequence to the virtual sequence. Empirical analyses reveal that among the three parameters, the starting temperature and steps can be set as constant, but the numbers of trials need to be large enough because it will decide the quality of the result sequence, which is proved by this figure. An unweighted dataset was applied to perform the test. The order of index fossils in the result sequence was checked and compared with the order of the virtual sequence. It is obvious that the order of species in the virtual sequence is robust, and the consistency finally reaches 100% if the number of calculations is large enough. The fit is based on eight data points (R^2 = 0.98).

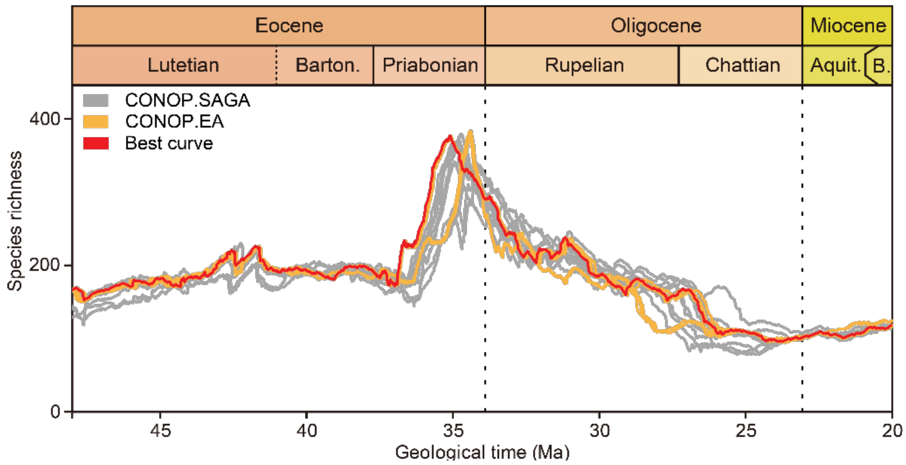

Fig. S7 | Richness curves based on various CONOP results. The grey lines represent 10 results using CONOP.SAGA. The orange lines show 10 results using CONOP.EA. The red line represents the most optimal curve.

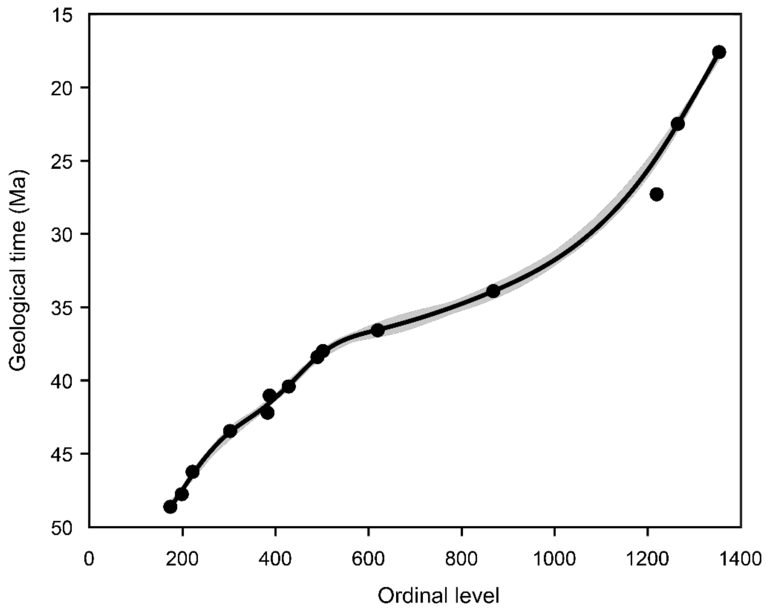

Fig. S8 | Age model of the most optimal composite sequence. The time scale comes from the GTS 2020. Solid line shows the smoothing-spline fit. Shaded area represents 95% confidence interval calculated from bootstrap with 1,000 replicates.

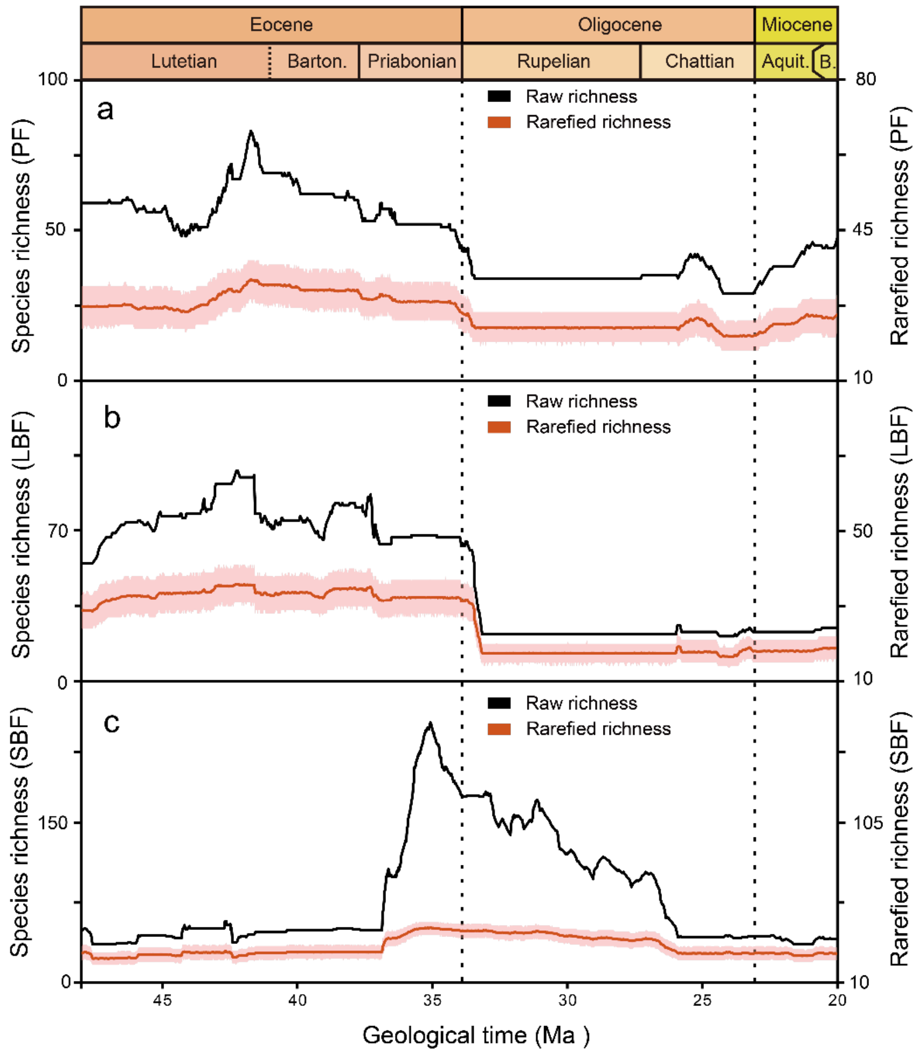

Fig. S9 | Rarefied richness for three foraminiferal groups. a, Planktonic foraminifera. b, Larger benthic foraminifera. c, Small benthic foraminifera. Sample size used in rarefaction is 50 species from observed sections and/or cores. The Solid lines represent point estimates from the original data. The shading represents the 95% confidence interval after repeating the rarefaction procedure 1,000 times.

Table S1 | Spearman's rank correlation among species richness time series data of three different foraminiferal groups.

	PF		LBF		SBF	
	ρ (95% CI)	P	ρ (95% CI)	P	ρ (95% CI)	P
PF	/	/	0.87 (0.82, 0.91)	8.89×10^{-44}	-0.28 (-0.43, -0.12)	8.89×10^{-4}
LBF	0.87 (0.82, 0.91)	8.89×10^{-44}	/	/	-0.22 (-0.37, -0.07)	0.009
SBF	-0.28 (-0.43, -0.12)	8.89×10^{-4}	-0.22 (-0.37, -0.07)	0.009	/	/

Notes: ρ is Spearman's rank correlation coefficient; P values are two-tailed; 95% CIs are percentile bootstrap intervals (2.5^{th}–97.5^{th} percentiles; B = 10,000). Very small P values are reported as $P < 2.2\times10^{-308}$.

Table S2 | Index fossils used for the construction of the composite sequence.

No.	Name
1	*Catapsydrax dissimilis*
2	*Globoquadrina dehiscens*
3	*Paragloborotalia kugleri*
4	*Paragloborotalia pseudokugleri*
5	*Paragloborotalia opima*
6	*Chiloguembelina cubensis*
7	*Ciperoella angulisuturalis*
8	*Turborotalia ampliapertura*
9	*Pseudohastigerina naguewichiensis*
10	*Hantkenina alabamensis*
11	*Globigerinatheka index*
12	*Globigerinatheka semiinvoluta*
13	*Morozovelloides crassatus*
14	*Orbulinoides beckmanni*
15	*Morozovella aragonensis*
16	*Globigerinatheka kugleri*
17	*Guembelitrioides nuttalli*
18	*Turborotalia frontosa*

Table S3 | Ten calculations using CONOP.SAGA.

No.	Parameters	Penalty	Time cost (hh:mm:ss)
1	500-700-2.4M	33692.3	3:03:28
2	500-700-2.4M	33859	3:02:13
3	500-700-2.4M	33876.1	3:02:36
4	500-700-2.4M	33925.6	3:02:27
5	500-700-2.4M	33758.6	3:03:07
6	500-700-4.8M	33655.9	6:08:13
7	500-700-4.8M	33654.7	6:06:49
8	500-700-4.8M	33659.1	6:03:16
9	500-700-4.8M	33628.8	6:03:43
10	500-700-4.8M	33659.3	6:01:42

Note: Computations are conducted on the Tianhe II Supercomputer with 4 nodes (256 CPU cores), and 1 TB of user file-system space. Parameters indicate those major settings used in the simulated annealing process, i.e., starting temperature, steps, and trials. For example, 500-700-2.4M means the starting temperature was set as 500°C, the cooling step was conducted 700 times, and the number of iteration calculations (trials) in each cooling step was 2,400,000.

Table S4 | Interpolated geochronologic ages for zonal boundaries (align with the stage boundaries if available) used in the calibration of the composite sequence.

No.	Fossil name	Type	Age (Ma, GTS 2012)	Age (Ma, GTS 2020)
1	*Catapsydrax dissimilis*	Top	17.59	17.53
2	*Globoquadrina dehiscens*	Base	22.48	22.43
3	*Chiloguembelina cubensis*	LCO	27.29	28.1
4	*Hantkenina alabamensis*	Top	33.9	33.9
5	*Morozovelloides crassatus*	Top	37.99	38.26
6	*Orbulinoides beckmanni*	Base	40.41	40.5
7	*Turborotalia frontosa*	Base	48.62	48.27

SI References

1 BouDagher-Fadel, M. K. Biology and evolutionary history of larger benthic foraminifera. In *Dev. Palaeontol. Stratigr.* **21**, 1-37 (Elsevier, 2008).

2 Kučera, M. Planktonic foraminifera as tracers of past oceanic environments. In *Proxies in Late Cenozoic Paleoceanography* (eds Hillaire-Marcel, C. & de Vernal, A.) *Dev. Mar. Geol.* **1**, 213–262 (Elsevier, 2007).

3 Keller, G., MacLeod, N. & Barrera, E. Eocene-Oligocene faunal turnover in planktic foraminifera and Antarctic glaciation. In *Eocene-Oligocene Climatic and Biotic Evolution* (eds Prothero, D. R. & Berggren, W. A.) 218–244 (Princeton Univ. Press, Princeton, 1992).

4 Hayes, A., Kucera, M., Kallel, N., Sbaffi, L. & Rohling, E. J. Glacial Mediterranean sea surface temperatures based on planktonic foraminiferal assemblages. *Quat. Sci. Rev.* **24**, 999-1016 (2005).

5 Thiede, J. A glacial Mediterranean. *Nature* **276**, 680-683 (1978).

6 Bé, A. W. H. Biology of planktonic foraminifera. In *Foraminifera: Notes for a Short Course* (ed. Broadhead, T. W.) *Stud. Geol.* **6**, 51–89 (Univ. Tennessee, Knoxville, 1982).

7 Bijma, J., Faber, W. W. & Hemleben, C. Temperature and salinity limits for growth and survival of some planktonic foraminifers in laboratory cultures. *J. Foraminifer. Res.* **20**, 95-116 (1990).

8 Kuroyanagi, A. et al. Effect of dissolved oxygen concentration on planktonic foraminifera through laboratory culture experiments and implications for oceanic anoxic events. *Mar. Micropaleontol.* **101**, 28-32 (2013).

9 Li, M., Lei, Y. L., Li, T. G. & Jian, Z. M. Impact of temperature on intertidal foraminifera: Results from laboratory culture experiment. *J. Exp. Mar. Biol. Ecol.* **520**, 151224 (2019).

10 Murray, J. W. *Ecology and applications of benthic foraminifera*. (Cambridge University Press, 2006).

11 Beavington-Penney, S. J. & Racey, A. Ecology of extant nummulitids and other larger benthic foraminifera: applications in palaeoenvironmental analysis. *Earth Sci. Rev.* **67**, 219-265 (2004).

12 Uthicke, S. & Altenrath, C. Water column nutrients control growth and C:N ratios of symbiont-bearing benthic foraminifera on the Great Barrier Reef, Australia. *Limnol. Oceanogr.* **55**, 1681-1696 (2010).

13 Saraswat, R. et al. Effect of salinity induced pH/alkalinity changes on benthic foraminifera: A laboratory culture experiment. *Estuar. Coast. Shelf Sci.* **153**, 96-107 (2015).
14 Dong, S. S., Lei, Y. L., Li, T. G., Jian, Z. M. Responses of benthic foraminifera to changes of temperature and salinity: Results from a laboratory culture experiment. *Sci. China Earth. Sci.* **62**, 459–472 (2019).
15 Pearson, P. N., Olsson, R. K., Huber, B. T., Hemleben, C. & Berggren, W. A. *Atlas of Eocene planktonic foraminifera*. (Cushman Foundation for Foraminiferal Research Lawrence, Kans, 2006).
16 Wade, B. S., Olsson, R. K., Pearson, P. N., Huber, B. T. & Berggren, W. A. *Atlas of Oligocene planktonic foraminifera*. (Cushman Foundation for Foraminiferal Research, 2018).
17 Firth, J. V., Eldrett, J. S., Harding, I. C., Coxall, H. K. & Wade, B. S. Integrated biomagnetochronology for the Palaeogene of ODP Hole 647A: Implications for correlating palaeoceanographic events from high to low latitudes. *Geological Society, London, Special Publications* **373**, 29-78 (2013).
18 Kaminski, M. A. & Ortiz, S. The Eocene-Oligocene turnover of deep-water agglutinated foraminifera at ODP site 647, southern Labrador Sea (North Atlantic). *Micropaleontology* **60**, 53-66 (2014).
19 Ortiz, S. & Kaminski, M. A. Record of deep-sea, benthic elongate-cylindrical foraminifera across the Eocene-Oligocene transition in the North Atlantic ocean (ODP Hole 647A). *J. Foraminifer. Res.* **42**, 345-368 (2012).
20 Grandjean, M. Gephi: Introduction to network analysis and visualisation. (2015).
21 Blondel, V. D., Guillaume, J. L., Lambiotte, R. & Lefebvre, E. Fast unfolding of communities in large networks. *J. Stat. Mech. Theory Exp.* **2008**, P10008 (2008).
22 Rojas, A., Patarroyo, P., Mao, L., Bengtson, P. & Kowalewski, M. Global biogeography of Albian ammonoids: a network-based approach. *Geology* **45**, 659-662 (2017).
23 Sadler, P. M. Quantitative biostratigraphy—achieving finer resolution in global correlation. *Annu. Rev. Earth Planet. Sci.* **32**, 187-213 (2004).
24 Fan, J. X. et al. A high-resolution summary of Cambrian to Early Triassic marine invertebrate biodiversity. *Science* **367**, 272-277 (2020).
25 Raffi, I. et al. The Neogene Period. In *Geologic time scale 2020* (eds Gradstein, F. M., Ogg, J. G., Schmitz, M. D. & Ogg, G. M.) 1141-1215 (Elsevier, 2020).

26 Speijer, R. P., Pälike, H., Hollis, C. J., Hooker, J. J. & Ogg, J. G. The Paleogene Period. In *Geologic time scale 2020* (eds Gradstein, F. M., Ogg, J. G., Schmitz, M. D. & Ogg, G. M.) 1087-1140 (Elsevier, 2020).

27 Wade, B. S., Pearson, P. N., Berggren, W. A. & Pälike, H. Review and revision of Cenozoic tropical planktonic foraminiferal biostratigraphy and calibration to the geomagnetic polarity and astronomical time scale. *Earth Sci. Rev.* **104**, 111-142 (2011).

28 Agterberg, F. P., Da Silva, A-C. & Gradstein, F. M. Geomathematical and statistical procedures. In *Geologic time scale* (eds Gradstein, F. M., Ogg, J. G., Schmitz, M. D. & Ogg, G. M.) 402-525 (2020).

29 Alroy, J. et al. Effects of sampling standardization on estimates of Phanerozoic marine diversification. *Proc. Natl. Acad. Sci. U.S.A.* **98**, 6261-6266 (2001).

30 Benson, R. B. & Butler, R. J. Uncovering the diversification history of marine tetrapods: ecology influences the effect of geological sampling biases. *Geological Society, London, Special Publications* **358**, 191-208 (2011).

31 Kidwell, S. M. & Holland, S. M. The quality of the fossil record: Implications for evolutionary analyses. *Annu. Rev. Ecol. Evol. Syst.* **33**, 561-588 (2002).

32 Lloyd, G. T., Pearson, P. N., Young, J. R. & Smith, A. B. Sampling bias and the fossil record of planktonic foraminifera on land and in the deep sea. *Paleobiology* **38**, 569-584 (2012).

33 Vilhena, D. A. & Smith, A. B. Spatial bias in the marine fossil record. *PLoS One* **8**, e74470 (2013).

34 Foote, M. Rarefaction analysis of morphological and taxonomic diversity. *Paleobiology* **18**, 1-16 (1992).

35 Foote, M., Miller, A. I. *Principles of paleontology*. (W. H. Freeman, 2007).

36 Chen, Q., Fan, J. X. & Melchin, M. J. Methods for paleobiodiversity measurement and case studies of their applicability. *Acta Palaeontol. Sinica* **51**, 445-462 (2012).

37 Foote, M. Origination and extinction components of taxonomic diversity: general problems. *Paleobiology* **26**, 74-102 (2000).

38 Pälike, H. et al. A Cenozoic record of the equatorial Pacific carbonate compensation depth. *Nature* **488**, 609-614 (2012).

39 Westerhold, T. et al. An astronomically dated record of Earth's climate and its predictability over the last 66 million years. *Science* **369**, 1383-1387 (2020).

40 Miller, K. G. et al. Cenozoic sea-level and cryospheric evolution from deep-sea geochemical and continental margin records. *Sci. Adv.* **6**, eaaz1346 (2020).

41 Auderset, A. et al. Enhanced ocean oxygenation during Cenozoic warm periods. *Nature* **609**, 77-82 (2022).

42 Cramer, B. S., Miller, K. G., Barrett, P. J. & Wright, J. D. Late Cretaceous–Neogene trends in deep ocean temperature and continental ice volume: Reconciling records of benthic foraminiferal geochemistry (δ18O and Mg/Ca) with sea level history. *J. Geophys. Res. Oceans* **116**, C12023 (2011).

43 Peters, S. E., Kelly, D. C. & Fraass, A. J. Oceanographic controls on the diversity and extinction of planktonic foraminifera. *Nature* **493**, 398-401 (2013).

44 Lazarus, D., Barron, J., Renaudie, J., Diver, P. & Türke, A. Cenozoic planktonic marine diatom diversity and correlation to climate change. *PLoS One* **9**, e84857 (2014).
