# Supplementary material for: Complex marine ecological response during the Eocene-Oligocene revealed by global foraminiferal record
Source: Nat Commun. 2026 Mar 14;17:3954. doi: 10.1038/s41467-026-70541-w (PMC13133226; doi:10.1038/s41467-026-70541-w)
Supplement: Supplementary file 2 — Descriptions of Additional Supplementary Files [file 41467_2026_70541_MOESM2_ESM.pdf]

Descriptions of Additional Supplementary Files

Supplementary Data 1. Fossil occurrence records used in this study.
This file contains occurrence-level records (one row per record) compiled and curated for the global foraminiferal dataset stored in the OneStratigraphy database. Columns include taxon information and taxonomy notes, section identifiers and within-section position (e.g., depth), locality metadata, stratigraphic descriptors, and bibliographic provenance (reference identifiers and citations). Fields are provided as recorded/curated in the compilation.

Supplementary Data 2. Inventory of sections and associated metadata used in this study.
This file provides a section-level inventory (one row per section) compiled and curated for this study. Columns include section names and identifiers, dataset descriptors (e.g., data source), entry/provenance fields, and bibliographic information (authors, year, and citation). Fields are provided as recorded/curated in the compilation.
