# Supplementary material for: Complex marine ecological response during the Eocene-Oligocene revealed by global foraminiferal record
Source: Nat Commun. 2026 Mar 14;17:3954. doi: 10.1038/s41467-026-70541-w (PMC13133226; doi:10.1038/s41467-026-70541-w)
Supplement: Supplementary file 5 — Reporting Summary [file 41467_2026_70541_MOESM5_ESM.pdf]

nature portfolio

Corresponding author(s): Junxuan Fan

Last updated by author(s): Feb 19, 2026

Reporting Summary

Nature Portfolio wishes to improve the reproducibility of the work that we publish. This form provides structure for consistency and transparency in reporting. For further information on Nature Portfolio policies, see our Editorial Policies and the Editorial Policy Checklist.

Statistics

For all statistical analyses, confirm that the following items are present in the figure legend, table legend, main text, or Methods section.

n/a	Confirmed	
☐	☒	The exact sample size (*n*) for each experimental group/condition, given as a discrete number and unit of measurement
☐	☒	A statement on whether measurements were taken from distinct samples or whether the same sample was measured repeatedly
☐	☒	The statistical test(s) used AND whether they are one- or two-sided<br>*Only common tests should be described solely by name; describe more complex techniques in the Methods section.*
☒	☐	A description of all covariates tested
☒	☐	A description of any assumptions or corrections, such as tests of normality and adjustment for multiple comparisons
☐	☒	A full description of the statistical parameters including central tendency (e.g. means) or other basic estimates (e.g. regression coefficient) AND variation (e.g. standard deviation) or associated estimates of uncertainty (e.g. confidence intervals)
☐	☒	For null hypothesis testing, the test statistic (e.g. *F*, *t*, *r*) with confidence intervals, effect sizes, degrees of freedom and *P* value noted<br>*Give P values as exact values whenever suitable.*
☒	☐	For Bayesian analysis, information on the choice of priors and Markov chain Monte Carlo settings
☒	☐	For hierarchical and complex designs, identification of the appropriate level for tests and full reporting of outcomes
☐	☒	Estimates of effect sizes (e.g. Cohen's *d*, Pearson's *r*), indicating how they were calculated

Our web collection on statistics for biologists contains articles on many of the points above.

Software and code

Policy information about availability of computer code

Data collection	no software was used for data collection.
Data analysis	The code was written in C++ and Python programming language. The software is provided as the following link: Dryad (July 29, 2025 version; https://doi.org/10.5061/dryad.jh9w0vtk5) and Zenodo (Version v2; https://doi.org/10.5281/zenodo.16336191).

For manuscripts utilizing custom algorithms or software that are central to the research but not yet described in published literature, software must be made available to editors and reviewers. We strongly encourage code deposition in a community repository (e.g. GitHub). See the Nature Portfolio guidelines for submitting code & software for further information.

Data

Policy information about availability of data

All manuscripts must include a data availability statement. This statement should provide the following information, where applicable:

- Accession codes, unique identifiers, or web links for publicly available datasets
- A description of any restrictions on data availability
- For clinical datasets or third party data, please ensure that the statement adheres to our policy

All data to reproduce this study are available in Dryad (July 29, 2025 version; https://doi.org/10.5061/dryad.jh9w0vtk5) and Zenodo repositories (Version v2; https://doi.org/10.5281/zenodo.16336191). The global foraminiferal data were derived from the OneStratigraphy database (http://onestratigraphy.ddeworld.org), an open-source database. Source data are provided with this paper. The deposited datasets are publicly available with no access restrictions.

Research involving human participants, their data, or biological material

Policy information about studies with human participants or human data. See also policy information about sex, gender (identity/presentation), and sexual orientation and race, ethnicity and racism.

Reporting on sex and gender	N/A
Reporting on race, ethnicity, or other socially relevant groupings	N/A
Population characteristics	N/A
Recruitment	N/A
Ethics oversight	N/A

Note that full information on the approval of the study protocol must also be provided in the manuscript.

Field-specific reporting

Please select the one below that is the best fit for your research. If you are not sure, read the appropriate sections before making your selection.

☒ Life sciences ☐ Behavioural & social sciences ☐ Ecological, evolutionary & environmental sciences

For a reference copy of the document with all sections, see nature.com/documents/nr-reporting-summary-flat.pdf

Life sciences study design

All studies must disclose on these points even when the disclosure is negative.

Sample size	We assembled a global foraminifera dataset comprising approximately 40,000 fossil occurrences of 1,269 species from 161 stratigraphic records (drill cores and outcrops), residing in the OneStratigraphy database (http://onestratigraphy.ddeworld.org/) and Dryad (July 29, 2025 version; https://doi.org/10.5061/dryad.jh9w0vtk5). Richness patterns were quantified both with and without sub-sampling. Details of our sub-sampling procedure are provided in the methods and supplementary information.
Data exclusions	No data were excluded from our study, unless samples were deemed to have been subject to open nomenclature (spp., aff., ?, etc.) and typo error, which could compromise the accuracy of biostratigraphic correlation based on species first/last appearances and distort understanding of species richness.
Replication	We analysed foraminiferal diversity patterns by applying computation on supercomputer 20 times (10 for two algorithms, respectively) , in order to assess the robustness of our pattern. All replications successfully show comparable results, and details are provided in the methods and supplementary information.
Randomization	We employed subsampling to estimate richness using bootstrap replicates, detailed in the methods.
Blinding	Blinding was not relevant to this study, since no human subjects were involved

Reporting for specific materials, systems and methods

We require information from authors about some types of materials, experimental systems and methods used in many studies. Here, indicate whether each material, system or method listed is relevant to your study. If you are not sure if a list item applies to your research, read the appropriate section before selecting a response.

Materials & experimental systems

n/a	Involved in the study	
☒	☐	Antibodies
☒	☐	Eukaryotic cell lines
☐	☒	Palaeontology and archaeology
☒	☐	Animals and other organisms
☒	☐	Clinical data
☒	☐	Dual use research of concern
☒	☐	Plants

Methods

n/a	Involved in the study	
☒	☐	ChIP-seq
☒	☐	Flow cytometry
☒	☐	MRI-based neuroimaging

Palaeontology and Archaeology

Specimen provenance	We quantified high-resolution temporal patterns in species richness using approximately 40,000 fossil occurrences of 1,269 species from 161 stratigraphic records (drill cores and outcrops) from OneStratigraphy database. Specimen locality information in OneStratigraphy database was derived primarily from references based on samples from field works and ocean drilling projects. Foraminifera are single-celled protists that live in the ocean.
Specimen deposition	All data can be accessed through Dryad (July 29, 2025 version; https://doi.org/10.5061/dryad.jh9w0vtk5), and through the open access database, OneStratigraphy (http://onestratigraphy.ddeworld.org/).
Dating methods	Age calibration is based on planktonic foraminiferal and magneto-chronology. The geologic age is following GTS 2020 (Ogg, 2020; Raffi et al., 2020; Speijer et al., 2020).

☒ Tick this box to confirm that the raw and calibrated dates are available in the paper or in Supplementary Information.

Ethics oversight	None.

Note that full information on the approval of the study protocol must also be provided in the manuscript.

Plants

Seed stocks	N/A
Novel plant genotypes	N/A
Authentication	N/A
